# Immune Microenvironment in Tumor Progression: Characteristics and Challenges for Therapy

**DOI:** 10.1155/2012/608406

**Published:** 2012-08-08

**Authors:** Valerie Chew, Han Chong Toh, Jean-Pierre Abastado

**Affiliations:** ^1^Agency for Science, Technology and Research (A∗STAR), Biopolis, Singapore Immunology Network (SIgN), Singapore 138648; ^2^Department of Medical Oncology, National Cancer Centre, Singapore

## Abstract

The tumor microenvironment plays a critical role in cancer development, progression, and control. The molecular and cellular nature of the tumor immune microenvironment influences disease outcome by altering the balance of suppressive versus cytotoxic responses in the vicinity of the tumor. Recent developments in systems biology have improved our understanding of the complex interactions between tumors and their immunological microenvironment in various human cancers. Effective tumor surveillance by the host immune system protects against disease, but chronic inflammation and tumor “immunoediting” have also been implicated in disease development and progression. Accordingly, reactivation and maintenance of appropriate antitumor responses within the tumor microenvironment correlate with a good prognosis in cancer patients. Improved understanding of the factors that shape the tumor microenvironment will be critical for the development of effective future strategies for disease management. The manipulation of these microenvironmental factors is already emerging as a promising tool for novel cancer treatments. In this paper, we summarize the various roles of the tumor microenvironment in cancer, focusing on immunological mediators of tumor progression and control, as well as the significant challenges for future therapies.

## 1. Introduction

The tumor microenvironment consists of cancer cells, stromal tissue, and extracellular matrix. The immune system is an important determinant of the tumor microenvironment. Indeed, the complex interplay between cancer cells and the host immune response has been extensively investigated in the past few decades. Several immunological deficiencies have been linked with enhanced tumor development in mouse models as well as in humans [1, 2]. The higher incidence of cancers in transplant patients receiving long-term immunosuppressive treatment is well documented [3–5]. Similarly, mice with compromised immune functions due to genetic modifications develop more tumors [6–9]. It is now well recognized that effective tumor surveillance by the immune system is critical to maintain homeostasis in the host.

Despite exerting a key role in host protection, tumor surveillance by the immune system may eventually fail. As described in the three “Es” of cancer immunoediting, tumor cells are initially eliminated by the immune system before becoming clinically detectable. This is then followed by an equilibrium phase, where a selection process for less immunogenic tumor variants take place until the tumors finally “escape” the immune surveillance [10, 11]. On the other hand, the persistent inflammation associated with chronic infections may also encourage new tumor formation [12]. Colorectal, hepatocellular, cervical, and gastric carcinomas are strongly associated with underlying chronic inflammatory responses [13, 14]. Expression of various immunological gene products during ongoing inflammation thus appears to create a favorable microenvironment for tumor growth and progression [10, 14].

Interestingly, recent large scale genomics studies conducted in cancer patients have revealed that the profile of the tumor microenvironment, and in particular the acute inflammation of host tissues, is linked with a better patient prognosis [15–17]. The tumor often benefits from an immunocompromised microenvironment in which regulatory immune components predominate. In contrast, patients who maintain active, proinflammatory immune responses within the tumor microenvironment achieve better outcomes [18, 19]. In the current paper, we focus on the role of host immune components in shaping the tumor microenvironment and the subsequent impact on disease progression.

## 2. Characteristics of the Tumor**** Microenvironment

The tumor microenvironment is made up of several important components including the tumor parenchyma cells, fibroblasts, mesenchymal cells, blood, and lymph vessels, as well as tumor infiltrating immune cells, chemokines, and cytokines [20]. These numerous and varied constituents fulfill the definition of a complex system, whereby the interactions between the components are multilevel, multiscale, and consist of nonlinear dynamics [21]. Each of these components can make important contributions to tumor development and progression.

Among these nonimmune components, tumor-associated fibroblasts are responsible for the formation and remodeling of the extracellular matrix and constitute a source of growth factor which promotes the growth of carcinoma cells [22]. The formation of new blood vessels is critical for tumor progression as the mass grows bigger [23], while existing blood and lymphatic vessels may act as routes for local invasion and distant metastasis [24, 25]. Many studies have shown that the density of blood vessels and the production of factors that stimulate blood vessel formation, including vascular endothelial growth factor (VEGF), platelet-derived growth factor (PDGF), and matrix metalloproteinases (MMPs), contribute to the spread of tumor cells and predict poor patient survival [24]. Other host cell lineages including mesenchymal stem cells not only form new carcinoma cells, but are also able to differentiate into the various cell types required to drive angiogenesis during cancer progression [26].

On the other hand, the immune components of tumor microenvironment have gained attention in the recent decades for their critical role in tumorigenesis and tumor control. Tumor-infiltrating immune cells including myeloid-derived suppressor cells (MDSC), tumor-associated macrophages (TAM), and cytotoxic lymphocytes are critical determinants of cancer outcomes. Many studies have shown that increased densities of MDSC and TAM promote tumor progression via multiple suppressive mechanisms [27, 28]. In contrast, the presence of cytotoxic lymphocytes within the tumor microenvironment is associated with a good prognosis in numerous cancers [15, 18, 29].

Other immune components of the tumor microenvironment, including chemokines and cytokines, may also alter the local balance of proregulatory and antitumor immune responses [30, 31]. Danger signals such as heat-shock proteins, nucleic acids, and HMGB1 released from transformed, dying, or dead tumor cells in the microenvironment are sensed by innate immunity components such as the toll-like receptors (TLRs) and can activate antitumor immune responses [32, 33].

## 3. The Role of the Immune Microenvironment in Tumor Control during the Initial Elimination/Equilibrium Phase

From many studies over the past decades, it is clear that the immune system plays a critical role in surveillance against tumor development. Immunodeficient mice defective in interferon (IFN)-*γ*, perforin, T cell, or NK cell functions develop tumors spontaneously [6–9]. For instance, the role of perforin in T and NK-cell-mediated cytotoxicity against injected tumor cells has been clearly established using for example perforin-deficient mice [9] but their exact role against established tumors is debated [34]. In humans, higher incidence of tumors can be observed in individuals with compromised immune systems (i.e., AIDS patients) [35] and in transplant recipients receiving long-term immunosuppressive therapy [3–5]. Transmission of donor melanoma to organ recipients by transplantation has also been reported [36].

Multiple lineages of immune cells are involved in antitumor responses. It has long been established that NK cells are able to kill tumor cells in various cancer models [37, 38]. Similarly, cytotoxic T lymphocytes can detect “abnormal” tumor antigens expressed on carcinoma cells and target those cells for destruction [39]. Antigen-presenting cells, and dendritic cells in particular, process and present tumor-derived antigens in the context of MHC class I molecules to activate CD8^+^ T cells via a mechanism known as cross-presentation [40]. Apart from direct killing of carcinoma cells, the activated CD8^+^ T cells may also inhibit angiogenesis by secreting IFN-*γ* [41]. B cell activation and differentiation into antibody-producing plasma cells as well as T- and B-cell memory are each important components of long lasting immune surveillance in cancer vaccines [42].

The period during which tumor cells are constantly being killed and controlled by the immune system is referred to as the “equilibrium phase.” The tumor can stay dormant for long periods of time until the microenvironment becomes permissive for growth [43]. This tumor dormancy is effectively recapitulated in our spontaneous melanoma mouse model wherein tumor cells disseminate early but remain dormant at remote metastatic sites [44]. Dormancy is partly controlled by cytotoxic CD8^+^ T cells, since depletion of CD8^+^ T cells results in faster outgrowth of visceral metastases [44]. However, the continuous control of tumor cells exerts a selective pressure which eventually favors the more aggressive tumor cells. For example, these tumor cell variants may mutate surface antigens in order to become less immunogenic via a process termed “immunoediting.” The equilibrium phase will thus eventually reach a state of exhaustion when the more aggressive and less immunogenic tumor cells are able to “escape” immune surveillance.

## 4. The Role of the Immune Microenvironment in Promoting Tumor Development and Progression: The “Escape” Phase

Tumor development and progression are influenced by modifications to tumor parenchymal cells or their microenvironment. One important mechanism of escape from immune surveillance is the selection of poorly immunogenic tumor cells [45]. Alternatively, modification of the microenvironment may also result in the acquisition of a “procancer” profile that encourages tumor outgrowth. These procancer modifications include the expression of antiapoptotic molecules which prevent tumor cell death [46]; growth factors which encourage tumor outgrowth [47], and immunosuppressive mediators such as VEGF, transforming growth factor-*β* (TGF-*β*), interleukin (IL)-10, indoleamine 2,3-dioxygenase (IDO), and programmed cell death-ligand 1 (PD-L1) which suppress antitumor immunity [48, 49]. Toll-like receptor (TLR) pathways such as TLR4 activation on tumor cells have also been shown to directly stimulate tumor growth [50]. Furthermore, as a result of imbalances between pro- and antiangiogenic factors, the microvasculature formed within the tumor microenvironment is often leaky and dysfunctional [51], which can limit T-cell infiltration and drug diffusion into the tumor. The tumor microenvironment is further shaped by resident leukocytes and the ongoing recruitment of different immune cell subsets. For example, the recruitment of regulatory T cells (Treg) and myeloid-derived suppressor cells (MDSC) contributes to immunosuppression within the tumor microenvironment [27, 52]. MDSC for instance act on multiple levels to inhibit naïve T-cell proliferation and differentiation, to block T effector cell functions, and to induce Tregs via the expression of IL-10 and TGF-*β* [27]. TAM (with a unique M2-like phenotype) have similarly been shown to correlate with poor prognosis in various cancers due to their immunosuppressive and angiogenic or lymphangiogenic properties [28]. The contribution of other leukocyte subsets to shaping the tumor microenvironment is less clear. While the role for Th17 cells in cancer is rather controversial [53], investigators have reported that these cells are associated with a poor prognosis in colorectal cancer [29]. Previous reports have even implicated B cells in enhanced tumor metastasis [54, 55]. On the other hand, the tumor microenvironment has been reported to prevent dendritic cell maturation hence making them incapable of functioning as effective antigen-presenting cells (APC) to trigger antitumor immunity [56].

Inflammation has been implicated in the development of cancers since the seminal observation made by Virchow in 1863 [13, 14] that chronic inflammation creates a microenvironment conducive to tumorigenesis. The inflammation associated with chronic infections such as *Helicobacter pylori* or hepatitis B virus promotes the respective development of gastric and liver cancers [57, 58]. Chronic inflammation-associated mechanisms of tumorigenesis include cellular transformation, proliferation, invasion, angiogenesis, chemoresistance, metastasis, and inhibition of apoptosis [13, 59]. Proinflammatory cytokines such as IL-6, IL-1*α*, and IL-8, as well as various chemokines, are known to favor tumor growth and progression [13, 14]. The inappropriately named tumor necrosis factor (TNF)-*α* has also been linked to several aspects of tumorigenesis including cellular transformation, proliferation, invasion, and metastasis [13]. The role of IL-6 and STAT-3 as antiapoptotic factors in various cancers is also well recognized [60]. Chemokines such as CXCL1 and CXCL8 are able to enhance tumor cell proliferation [61]; CXCL5 and CXCL12 attract neutrophils and MDSC [62], while CXCL12 promotes the migration of tumor cells that express the cognate receptor CXCR4 [63]. Many of these immunological mediators are regulated by transcription factor NF-*κ*B, which is constitutively active in many cancers and is inducible by various carcinogens including viruses [13, 64].

Tumor metastasis is the primary cause of cancer-related death [65]. Epithelial-to-mesenchymal transition (EMT) of cancer cells is associated with enhanced cell migration, local invasion, and distant metastasis, while expression of EMT markers correlates with poor prognosis [66]. EMT is a common process in early embryogenesis and carcinoma progression [67]. During EMT, the carcinoma cells undergo morphological changes that confer enhanced motility and reduced intercellular adhesion which enable local invasion and distant metastasis [68]. Our recent study in a spontaneous melanoma model showed that tumor recruitment of MDSC promotes EMT [69]. In particular, we found that granulocytic (G)-MDSC induce EMT *in vitro* and *in vivo* via multiple pathways that involve TGF-*β*1, epidermal growth factor (EGF), and hepatocyte growth factor (HGF) [69]. Other immune cells such as activated CD8^+^ T cells [70] and macrophages [71] have also been shown to stimulate EMT in tumor-bearing mice. Together, these data emphasize the intimate relationship between host immune responses and the microenvironment in shaping tumor development and progression.

## 5. The Role of the Immune Microenvironment in Controlling Tumor Progression of**** Established Tumors

Even as the immune system fails to control tumor formation, the immune response within the microenvironment of established tumors remains an important factor in determining the outcome of cancer. Regression of established liver tumors by induction of CD8^+^ T-cell responses with peptide-based immunotherapy was reported in several mouse models [72, 73]. Recent genomics studies in various human tumors including breast cancer have identified immunological parameters as important determinants of disease outcome [16, 17, 19]. Several studies have underlined the importance of the tumor microenvironment on the clinical evolution of HCC [19, 74]. Our studies revealed an association between the expression of intratumoral proinflammatory genes and superior patient survival [15, 19]. In 172 HCC patients, we demonstrated that a 14-gene immunological signature is predictive of patient survival, especially at the early stages of the disease [15]. These 14 immune genes encode chemokines CXCL10, CCL5, and CCL2; cytokines IFNG, TNF, and IL6; pattern recognition receptors TLR3 and TLR4; T cell markers CD8A and TBX21, and NK cell marker NCR3. In this study, we showed that IFN-*γ* and TLR3 ligand-induced intra-tumor chemokine expression promotes infiltration by cytotoxic T cells and NK cells to enhance tumor cell apoptosis and reduce tumor cell proliferation [15]. The immune microenvironment of noncancerous hepatic tissues has also been shown to impact on the development of venous metastases in HCC patients [75].

A proinflammatory phenotype combined with tumor infiltration by cytotoxic lymphocytes is associated with a better prognosis in various cancers [15, 18]. Tumor infiltration by T cells has now been linked with favorable prognosis in colorectal cancer [76], melanoma [77], breast cancer [78], ovarian cancer [79], and lung cancer [80]. Recent studies in liver and breast cancers have identified an important correlation between the densities and distribution of T and B cells with a favorable prognosis [81, 82]. Our own study in HCC revealed a correlation between superior patient survival and the intratumor densities of T cells and NK cells [15].

It is important to appreciate that tumor infiltration by cytotoxic lymphocytes is often orchestrated by chemokines expressed within the tumor microenvironment. In HCC, we demonstrated that stimulation with cytokines in conjunction with TLR activation can promote inflammation and chemokine production in tumors [15]. Chemokine-mediated tumor infiltration by cytotoxic lymphocytes has also been demonstrated by other investigators [83, 84]. In a cutaneous melanoma model, we further showed that chemotherapy could induce intra-tumor expression of chemokines that favored T-cell infiltration and tumor control [85]. In contrast, several studies have highlighted the key role played by chemokines during metastasis, particularly among tumor cells that express chemokines receptors CXCR3 and CXCR4 [86, 87]. The role of the proinflammatory microenvironment in tumor control therefore appears to be context dependent and will require further detailed investigation.

## 6. Challenges in Tumor Immunotherapy

**Figure 1:**
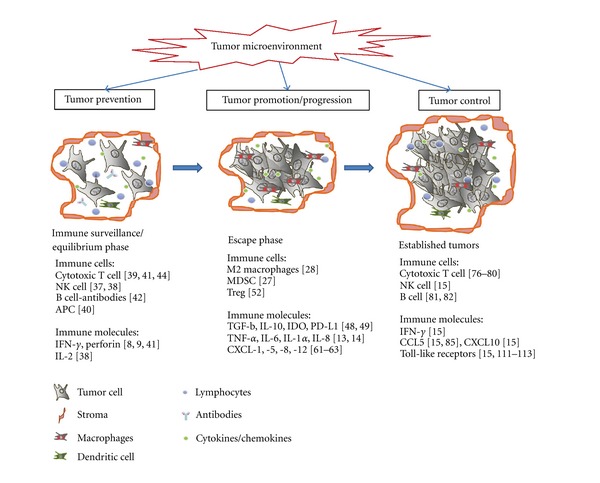
Multiple roles of the immune microenvironment during tumor development. The immune system initially eliminates tumor cells via cytotoxic T cell and NK cell killing mechanisms (immune surveillance). This is achieved with the help of antigen-presenting cells (APC) such as the dendritic cells, antibodies expressed by B cells, and inflammatory cytokines including IFN-*γ* and IL-2 which activate the local immune response. However, with the progressive accumulation of tumor cell mutations and modifications to the microenvironment, the tumor cells can eventually “escape” from immune surveillance. Multiple lineages of immune cells including myeloid-derived suppressor cells (MDSC), tumor-associated macrophages (TAM), and regulatory T cells (Treg), as well as various immune mediators such as TNF-*α*, IL-6, CXCL-1, CXCL-5, VEGF, and MMP, are responsible for shaping a favorable microenvironment for tumor growth. Recent findings also show that the immune response continues to play an important role in established tumors via mechanisms that involve cytotoxic T cells and NK cells, as well as IFN-*γ*, CCL5, CXCL10, and toll-like receptors.

Given the complex roles of the immunological microenvironment in tumor immunity (Figure 1), developing methods for targeting the relevant effector molecules or pathways for cancer treatment remains challenging. Indeed, the limited success of cancer immunotherapy to date can primarily be attributed to three main factors: (1) poor host responses towards tumor antigens, (2) low infiltration of effector cells into solid tumors, and (3) the intrinsically immunosuppressive tumor microenvironment. Tipping the balance of immune responses from tumor protection towards tumor rejection seems to be key for effective cancer immunotherapy [88–90]. Manipulation of the tumor microenvironment will therefore be an important consideration for achieving optimal antitumor responses with future treatments.

Several cases of spontaneous regression associated with specific antitumor immune responses have been reported in various cancers [91–93]. Efforts to activate local adaptive immune responses in tumors have met with some success, and cell-based therapies such as adoptive T-cell transfer have shown convincing signs of efficacy in treating metastatic melanoma patients [94]. Recent developments in cancer immunotherapies have now also begun to explore the use of NK cells [95, 96]. In particular, strategies that employ tumor-specific monoclonal antibodies (mAbs) and mAb-cytokine fusion proteins (immunocytokines, ICs) designed to augment NK-mediated killing have shown promising results in preclinical and some clinical settings [97].

Cancer vaccines aim to induce immune responses against tumor-associated antigens and several such vaccines are currently under development to treat various cancers [98, 99]. The first FDA-approved therapeutic cancer vaccine Provenge (Sipuleucel-T) provides modest but significant benefits in castrate-resistant prostate cancer [100]. However, the low immunogenicity of most tumor antigens represents a major difficulty in developing potent cancer vaccines. Intensive research will be needed to improve the specificity and effectiveness of these cancer vaccines. Furthermore, the immunosuppressive tumor microenvironment limits the effectiveness of the antitumor immune responses induced by these cancer vaccines [99]. Therefore, manipulation of the tumor microenvironment either by enhancing the antitumor activity or blocking the immunosuppressive pathways is among the strategies pursued for more effective tumor therapy.

Critical to accurately assessing efficacy of therapeutic cancer vaccines is to define appropriate clinical endpoints. The phase III evaluation of Provenge in castrate-resistant advanced prostate cancer revealed a significantly improved overall survival benefit without a significant improvement in progression-free survival (PFS). This implies that while tumor kinetics may have been favorably retarded by vaccine-induced antigen-specific immunity, the tumor growth may not have been rendered stable or regressed. Hence it will be a challenge to select objective response rates or even PFS as accurate measures of therapeutic cancer vaccine outcomes.

Alternatively, vaccines that aim to control the inflammation induced by chronic infections may serve as effective tumor prevention measures [101, 102]. One such example is the hepatitis B vaccination which has successfully reduced the incidence of liver cancer in Taiwan since being introduced in 1984 [103]. Vaccines against oncogenic human papilloma viruses (HPV) achieved similar success in preventing cervical cancer [104, 105]. Other cancer immunotherapies have included immunostimulatory cytokines such as IL-2 and IFN-*α* [106, 107], as well as antibodies against tumor antigens [108–110], for use as adjuvants in combination with chemotherapy or cancer vaccines. The use of toll-like receptor (TLR) ligands can trigger effective innate immune responses within tumors [111–113]. Some success has also been achieved with the application of TLR7 agonists in the treatment of skin carcinoma [113, 114]. The host response to endogenous danger signals could be yet another target for therapies that activate and maintain effective antitumor immunity [32, 115]. As intra-tumor expression of chemokines correlates with enhanced lymphocyte infiltration, transfection of chemokine cDNAs in murine tumor cells has shown promising tumor rejection in these preclinical models [116].

These recent advances in immunotherapy confirm that boosting the activity of tumor-infiltrating lymphocytes, which are reported to be exhausted in many cancers [117, 118], will be key to the development of the most effective treatments. Such strategies may include the blockade of immunosuppressive pathways including PD/PDL [119, 120], CTLA-4 [121, 122] and Cox 2 [123, 124], Treg depletion prior to vaccination [125, 126], or perhaps activation of the TLR pathway [112, 127]. For example, Ipilimumab, an antibody against CTLA-4, a key negative regulator of T cell responses, was recently approved by the FDA for the treatment of metastatic melanoma [121, 122]. Interestingly, the landmark clinical Phase III study of Ipilumumab in advanced melanoma also did not show a progression-free survival benefit even as the elusive significant overall survival benefit was achieved. These interventions enhance the effectiveness of therapies by pushing the immunological balance towards antitumor responses within the microenvironment of cancers [89, 90, 128].

Some cancer drugs that were initially developed to induce carcinoma cell death were later found to act on the tumor microenvironment. One such example is Imatinib mesylate (Gleevec), a tyrosine kinase inhibitor which was developed to inhibit tyrosine kinase BCR-ABL in chronic myeloid leukemia (CML). Gleevec was recently approved for the treatment of gastrointestinal stromal tumors (GIST) which exhibit a c-kit tyrosine kinase mutation. However, it was later shown that clinical responses to Imatinib correlated with the inhibition of immunosuppressive enzyme IDO and hence enhanced levels of T cell activation [129]. Interestingly, the class of small molecules that inhibit mTOR has recently been shown to exert antitumor activity by stimulating homeostatic proliferation of memory CD8^+^ T cells [130].

Cytotoxic or genotoxic agents which induce cellular stress or DNA damage could release danger signals that are sensed by toll-like receptors and activate innate immune responses [131]. Chemotherapeutic drugs have also been found to activate the immune system despite the prevailing view that these agents induce immunosuppressive effects. For example, low doses of cyclophosphamide inhibit Treg, and gemcitabine or 5-fluorouracil eliminate MDSC [132]. Cyclophosphamide, paclitaxel, doxorubicin, and vinblastine given at regular intervals normalize the tumor-associated vasculature, thereby facilitating the delivery of drugs and recruitment of T lymphocytes [133]. Gemcitabine can activate both the adaptive and humoral immunity to elicit meaningful antitumor responses in animal models [134]. In melanoma patients responding to dacarbazine, we also found that chemotherapy is able to induce intra-tumor expression of T cell and NK cell-attracting chemokines CXCL9, CXCL10, and CCL5, which was associated with improved survival [85]. It will be important therefore to develop future cancer drugs in the context of potential effects on the tumor microenvironment.

## 7. Conclusions

The immunological conditions in the tumor microenvironment are now well recognized to be a critical determining factor in tumor prevention, development, and progression. Considerable evidence has been provided by studies in various different cancers that the status of the tumor microenvironment is well correlated with disease outcome. The presence of particular immune cell types or molecules determines whether a pro- or antitumor immune response predominates within the microenvironment. The concept of switching the immune response from a tumor-promoting profile to a tumor-destructive profile is now widely regarded as key to the future success of cancer immunotherapies. The manipulation of immunological parameters which shape the tumor microenvironment may suffice to tip the balance of host responses towards effective immunity. Better understanding of the roles of immune cells and molecules in the tumor microenvironment will therefore be essential for the development of more effective novel treatments.
